# Optimal timing for the Modified Early Warning Score for prediction of short-term critical illness in the acute care chain: a prospective observational study

**DOI:** 10.1136/emermed-2022-212733

**Published:** 2024-04-26

**Authors:** Lars Ingmar Veldhuis, Merijn Kuit, Liza Karim, Milan L Ridderikhof, Prabath WB Nanayakkara, Jeroen Ludikhuize

**Affiliations:** 1 Emergency Department, Amsterdam UMC Locatie AMC, Amsterdam, The Netherlands; 2 Department of Anaesthesiology, Amsterdam UMC Locatie AMC, Amsterdam, The Netherlands; 3 Emergency Medicine, Amsterdam UMC - Locatie AMC, Amsterdam, The Netherlands; 4 Section Acute Medicine, Department of Internal Medicine, Amsterdam Universitair Medische Centra, Amsterdam, The Netherlands; 5 Department of Internal Medicine, Amsterdam UMC Locatie VUmc, Amsterdam, The Netherlands; 6 Department of Intensive Care, Haga Hospital, Den Haag, The Netherlands

**Keywords:** emergency department, emergency care systems, acute care, care systems, critical care

## Abstract

**Introduction:**

The Modified Early Warning Score (MEWS) is an effective tool to identify patients in the acute care chain who are likely to deteriorate. Although it is increasingly being implemented in the ED, the optimal moment to use the MEWS is unknown. This study aimed to determine at what moment in the acute care chain MEWS has the highest accuracy in predicting critical illness.

**Methods:**

Adult patients brought by ambulance to the ED at both locations of the Amsterdam UMC, a level 1 trauma centre, were prospectively included between 11 March and 28 October 2021. MEWS was calculated using vital parameters measured prehospital, at ED presentation, 1 hour and 3 hours thereafter, imputing for missing temperature and/or consciousness, as these values were expected not to deviate. Critical illness was defined as requiring intensive care unit admission, myocardial infarction or death within 72 hours after ED presentation. Accuracy in predicting critical illness was assessed using the area under the receiver operating characteristics curve (AUROC).

**Results:**

Of the 790 included patients, critical illness occurred in 90 (11.4%). MEWS based on vital parameters at ED presentation had the highest performance in predicting critical illness with an AUROC of 0.73 (95% CI 0.67 to 0.79) but did not significantly differ compared with other moments. Patients with an increasing MEWS over time are significantly more likely to become critical ill compared with patients with an improving MEWS.

**Conclusion:**

The performance of MEWS is moderate in predicting critical illness using vital parameters measured surrounding ED admission. However, an increase of MEWS during ED admission is correlated with the development of critical illness. Therefore, early recognition of deteriorating patients at the ED may be achieved by frequent MEWS calculation. Further studies should investigate the effect of continuous monitoring of these patients at the ED.

**WHAT IS ALREADY KNOWN ON THIS TOPIC**

- The Modified Early Warning Score (MEWS) is an effective tool to identify deteriorating patients in the acute care chain who might deteriorate.
- Although it is increasingly being implemented, the optimal timing for assessing the MEWS is unknown.

**WHAT THIS STUDY ADDS**

- This prospective multicentre study included 790 patients and found that MEWS measured at ED presentation had the highest accuracy in predicting the development of critical illness. However, the performance is moderate and not significantly better compared to MEWS based at other moments in the acute care chain.
- However, an increase in MEWS during the ED encounter is highly correlated with the development of critical illness.

**HOW THIS STUDY MIGHT AFFECT RESEARCH, PRACTICE OR POLICY**

- As clinical deterioration and subsequent development of critical illness is highly correlated with an increase of MEWS during the ED stay, we suggest further investigation on the value of continuous monitoring of these patients at the ED

## Introduction

Early recognition of the deteriorating patient is of vital importance to reduce the occurrence of serious adverse events (SAEs) including cardiopulmonary arrests, (delayed) intensive care unit (ICU) admissions and death. Prior research indicates that up to 80% of deteriorating patients show physiological abnormalities up to 24 hours before the event.^1–4^ Track and trigger systems, including the Early Warning Score (EWS) were developed to recognise the early signs of deterioration. These scoring systems are relatively simple models using the patients’ vital parameters to assess the degree of illness of the patient.

In general, the higher the EWS, the more likely it is that a patient is clinically deteriorating and subsequently becomes critically ill.^5^ This use of an EWS has proven to be efficient for detecting deteriorating patients on the wards.^6^ When a deteriorating patient is identified, the Medical Emergency Team can be consulted, and more appropriate care can be provided. The implementation of EWS-based systems can lead to a reduction in SAEs and reduced time to ICU admission in deteriorating patients.^7^

As the EWS-based system has been shown to be effective in general wards, the model has been increasingly implemented in other aspects of acute care, that is, the prehospital and ED settings.^8–11^ Several studies suggest that EWS can be useful in the entire acute care chain. Prior studies showed a MEWS performance in the ED setting of area under the receiver operating characteristics curve (AUROC) 0.65.^12^

However, it is unclear what moment in the acute care chain has the highest accuracy in predicting deterioration.

Timely interventions such as administration of antibiotics, and fluid challenges strongly affect vital parameters and overall survival.^13^ These interventions may stabilise the patient and prevent further deterioration, which influences the EWS.

The primary aim of this study was to determine at which time point, from the first moment of contact with the EMS to admission to a nursing ward, an EWS is most accurate in detecting a deteriorating patient. Although the National EWS is generally slightly more accurate compared with the Modified Early Warning Score (MEWS),^14^ we studied the performance of MEWS, as this is the tool regularly used in the Netherlands.

## Methods

### Study design and population

This was a prospective observational multicentre study, conducted at a university hospital, serving as a level 1 trauma centre with two locations. All adult patients (18 years and older) brought by ambulance to one of these two centres between 11 March and 28 October 2021, were included. Interhospital transfers and patients receiving prehospital cardiopulmonary resuscitation were excluded. Participants gave informed consent before taking part.

### Data collection

Data were collected by a researcher present during EMS presentation between 10:00 hours and 18:00 hours on workdays, as during this period most ambulances arrive at the EDs of both centres. As we recorded data up to 3 hours after ED presentation, data were obtained until 21:00 hours. Patient characteristics, including vital parameters measured at four time points were collected on paper forms: prehospital (recorded by the ambulance); at ED admission (±15 min); at 1 hour (±15 min); and at 3 hours (±30 min) after ED arrival. Three-day outcome was obtained from the electronic patient records. All obtained data were processed using a standardised data worksheet. Collected data were anonymously processed using an online data collection system (Castor eClinical Data Management).

### Endpoints and definitions

The primary outcome was the performance of MEWS in predicting critical illness for all four time points during which data were collected.

Secondary outcome was the association between the MEWS over time (ie, increase of MEWS 1 hour after ED admission compared with prehospital MEWS) and subsequent development of critical illness.

Critical illness was defined as mortality; ICU admission and/or myocardial infarction (as concluded by a cardiologist) all within 3 days after ED presentation.

**Figure 1:**
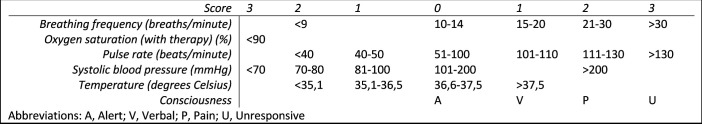
Modified Early Warning Score.

Primary and secondary outcome was assessed by investigating the electronic medical records on day 4 after the initial ED admission. MEWS was thereafter calculated using the vital parameters at each time point; see figure 1 for thresholds of the MEWS.

### Missing data

Previous studies have shown that the temperature and level of consciousness of patients generally remain constant from transportation by EMS to arrival at the ED.^13^ Therefore, in any cases where the temperature or level of consciousness of a patient was recorded prehospitally but missing at admission or vice versa, the recorded values for these parameters were used. MEWS was then calculated if a minimum of four out of six vital parameters was available with the one or two missing parameters considered normal. In choosing this method we acted on the assumption that diverging vital parameters would have been registered by the ED nurse. If more than two vital parameters were missing for a certain point in time, the MEWS at that time was not calculated. Patients for whom the MEWS could not be calculated were excluded from analysis for that specific point in time.

### Sensitivity analysis

Model performance was tested after excluding patients with SARS-CoV-2 infection, as patients with COVID-19 are known to have relatively stable vital parameters despite being critically ill (as compared with patients without COVID-19).^15^

#### Primary and secondary outcomes

The primary outcome was the performance of MEWS at different periods of time using the outcomes of developing critical illness (as defined above). The secondary outcome was whether an increase in MEWS over time was associated with becoming critically ill.

### Statistical analysis

Descriptive and statistical analysis was performed using SPSS V.22.0 (SPSS, Chicago, Illinois, USA). Non-normally distributed continuous variables were described as medians with IQRs and were compared with the Mann-Whitney *U* test. Categorical variables were described as numbers and percentages and were compared by Pearson’s χ^2^ test. The primary outcome was expressed as the AUROC of the MEWS for each time point. Also, for each MEWS between 0 and 5, sensitivity and specificity were calculated.

Using the AUROC derived from MEWS at the different time points, superiority in performance was assessed using the method of Hanley and McNeil.^16^ In general, the AUROC is characterised using standard terms, where AUROC 0.6–0.7 is considered a poor testing method, 0.7–0.8 is considered moderate, 0.8–0.9 is good and a test with an AUROC >0.9 is considered an excellent method.

A χ^2^ test was used to test whether an increase of MEWS over time had a higher incidence of becoming critically ill compared with a decreased or stable MEWS.

### Sample size calculation

For the sample size calculation, the previously reported performance (AUROC 0.65) of MEWS in the ED was used.^12^ For the primary outcome (the moment with the highest AUROC of MEWS) based on a 95% CI, 80% power and a 0.1 difference in MEWS, 114 patients were needed to test for statistically significant difference. These calculations were performed in nQuery tool for design of trials, link https://www.statsols.com/nquery.

### Patient and public involvement

Patients and/or the public were not involved in the design, or conduct, or reporting, or dissemination plans of this research.

## Results

Of the 790 patients included in this study, critical illness occurred in 90 patients (11.4%). Prehospital alert calls to the ED were made significantly more often for critically ill patients (88.9% vs 69.4%, p<0.001). Additionally, these patients were assessed more often in either the resuscitation or trauma bay (table 1).

**Table 1:** Patient characteristics

Patient characteristic	Critical illness <72 hours	Non-critical illness <72 hours	P value
Number of patients, n (%)	90	700	
Age, median (IQR)	67 (54 to 76)	66 (51 to 77)	0.812
Male, n (%)	56 (62.2)	381 (54.4)	0.162
Prehospital notification, n (%)	80 (88.9)	486 (69.4)	<0.001

Of the 90 critically ill patients, 41 patients were directly admitted from the ED to the ICU, 16 patients were initially admitted to the ward then went to the ICU, 15 died and 17 had a myocardial infarction, all within 72 hours after ED presentation. Prior to imputing for missing values, the number of complete MEWS values was limited (table 2). After imputing for missing values, the most complete moment of measurements was at ED arrival (94.8%).

**Table 2:** Complete MEWS before and after imputing

	Complete MEWS, n (%)	Breathing rate, n (%)	Saturation, n (%)	Systolic blood pressure, n (%)	Heart rate, n, (%)	AVPU, N (%)	Temperature, n (%)	Complete MEWS after imputing data, n (%)
Prehospital	**181 (22.6)**	364 (45.5)	613 (76.6)	674 (80.9)	638 (79.8)	638 (79.8)	521 (65.1)	**651 (81.4)**
At arrival	**283 (35.4)**	598 (74.8)	756 (94.5)	728 (91.0)	770 (96.3)	706 (88.3)	377 (47.1)	**758 (94.8)**
1 hour after arrival	**22 (2.8)**	474 (59.3)	517 (64.6)	355 (44.4)	613 (76.6)	181 (22.6)	59 (7.4)	**536 (67.0)**
3 hours after arrival	**25 (3.1)**	384 (48.0)	377 (47.1)	339 (42.4)	507 (63.4)	70 (8.8)	87 (10.9)	**443 (55.4)**

AVPU, alert, verbal, pain, unresponsive; MEWS, Modified Early Warning Score.

## Primary outcome

**Figure 2:**
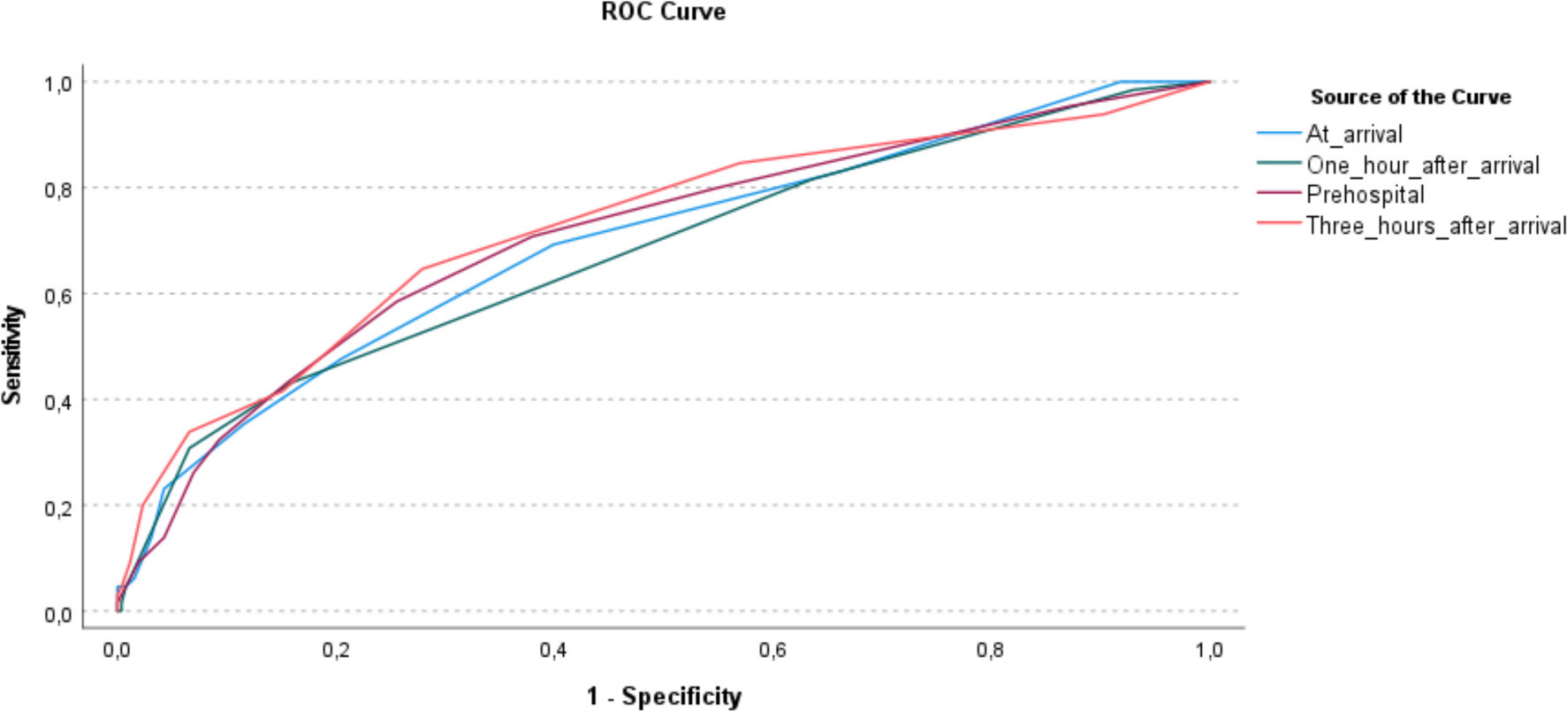
Receiver operating characteristics (ROC) curves for prediction of critical illness within 72 hours.

MEWS based on vital parameters measured at ED admission had the highest performance with an AUROC of 0.726 (table 3, figure 2). MEWS based on vital parameters measured 1 hour and 3 hours after ED admission had lower performance (table 4). The performance of MEWS measured at ED admission was not significantly superior compared with the other time points in predicting critical illness.

**Table 3:** AUROCs for the prediction of critical illness within 72 hours

	AUROC (95% CI) for predicting 3-day critical illness	P value for comparison with best performing time point	AUROC (95% CI) for predicting 3-day critical illness after excluding patients with COVID-19	P value for comparison including patients with COVID-19
Prehospital	0.711 (0.644 to 0.779)	0.757	0.715 (0.644 to 0.768)	0.939
At presentation	0.726 (0.666 to 0.786)	Reference	0.719 (0.656 to 0.783)	0.881
After 1 hour	0.674 (0.600 to 0.748)	0.289	0.683 (0.606 to 0.761)	0.866
After 3 hours	0.709 (0.640 to 0.777)	0.724	0.716 (0.644 to 0.788)	0.891

AUROC, area under the receiver operating characteristics curve.

**Table 4:** Sensitivity and specificity for cut-off points of MEWS

Timing	Cut-off points					
	0	1	2	3	4	5
Prehospital (n=642)						
Sensitivity 95% CI	100 (99.4 to 100)	94.3 (92.6 to 96.1)	72.9 (69.3 to 76.2)	62.9 (59.1 to 66.6)	52.9 (49.0 to 56.7)	38.6 (34.9 to 42.4)
Specificity 95% CI	0 (0 to 0.6)	20.6 (17.7 to 23.9)	53.1 (49.2 to 56.9)	69.1 (65.4 to 72.6)	79.9 (76.6 to 82.8)	88.6 (85.9 to 90.8)
Entrance (n=749)						
Sensitivity 95% CI	100 (99.5 to 100)	96.5 (94.9 to 97.6)	79.1 (76.0 to 81.9)	64.0 (60.5 to 67.4)	46.5 (43.0 to 50.1)	34.9 (31.6 to 38.4)
Specificity 95% CI	0 (0 to 0.5)	16.4 (13.9 to 19.2)	47.1 (43.6 to 50.7)	70.1 (66.7 to 73.3)	85.5 (82.8 to 87.8)	92.2 (90.1 to 93.9)
After 1 hour (n=529)						
Sensitivity 95% CI	100 (99.3 to 100)	95.8 (93.7 to 97.2)	74.6 (70.7 to 78.1)	57.7 (53.5 to 61.8)	39.4 (35.3 to 43.6)	32.4 (28.6 to 36.5)
Specificity 95% CI	0 (0 to 0.7)	10.9 (8.5 to 13.8)	41.3 (37.2 to 45.5)	70.1 (66.1 to 73.9)	86.0 (82.8 to 88.7)	94.8 (92.6 to 96.4)
After 3 hours (n=440)						
Sensitivity 95% CI	100 (99.1 to 100)	94.7 (92.2 to 96.4)	82.7 (78.9 to 86.0)	61.3 (56.7 to 65.7)	42.7 (38.2 to 47.4)	33.3 (29.1 to 37-8)
Specificity 95% CI	0 (0 to 0.9)	11.5 (8.8 to 14.8)	42.2 (37.7 to 46.9)	70.7 (66.3 to 74.8)	84.9 (81.3 to 87.9)	93.4 (90.7 to 95.4)

MEWS, Modified Early Warning Score.

Of the 790 patients, 82 had a proven SARS-CoV-2 infection. Excluding patients with a proven SARS-CoV-2 infection did not lead to a significant improvement of MEWS accuracy in predicting critical illness (table 3).

In addition, sensitivity and specificity were calculated for each threshold (table 4). For the MEWS measured at ED admission using a cut-off value of 3, sensitivity was 64.0% (95% CI 60.5% to 67.4%) and specificity was 70.1% (95% CI 66.7% to 73.3%).

### Secondary outcome

To estimate the influence of a change over time in MEWS (delta MEWS) on outcome, a χ^2^ test was performed. An increase in MEWS between the MEWS measured prehospitally and 1 hour after ED admission had an incidence of 25.7% of critical illness, while stable or decreasing MEWS had an incidence of 7.5%. This difference was significantly different (p<0.05) (see table 5).

**Table 5:** Changes in MEWS during admission and the development of critical illness

	Not critically ill	Critically ill
Stable/decreasing MEWS	248 (92.5%)	20 (7.5%)
Increased MEWS	133 (74.3%)	46 (25.7%)

MEWS, Modified Early Warning Score.

## Discussion

While many studies focus on the performance of EWS in either the prehospital or ED setting, little is known about the best timing to use it in the acute care chain.^8 17 18^ Therefore, this prospective multicentre study was performed to attempt to direct clinical practice to the best moment in the acute care chain to measure MEWS to identify subsequent development of critical illness in patients brought to the ED by ambulance. Although MEWS calculated based at presentation had the highest accuracy in predicting the development of critical illness, an AUROC of 0.726 was not significantly superior to MEWS measured prehospitally or 1 hour or 3 hours after ED presentation. Also, excluding patients with proven SARS-CoV-2 infection did not lead to an improvement in model performance. While the performance of MEWS found in this study in predicting critical illness is moderate, this was consistent with other studies.^19^

Our secondary outcome was to test the correlation between an increase of MEWS over time and the development of critical illness. Prior studies suggest that the trend of MEWS during the first hours of ED presentation may identify clinically deteriorating patients better compared with a single MEWS calculation.^5 10 20 21^ Our results indicate that an increase of MEWS between prehospital and at 1 hour after ED admission was significantly correlated with the development of critical illness, p=0.005. Therefore, we suggest that patients with an increasing MEWS during ED stay should be more intensively monitored and early consultation with the ICU consultant may be justifiable.

### Limitations

The study has several limitations which may reduce the generalisability of our data and have most likely influenced our results. First, the study ran during the summer months, so season-specific diseases may have occurred. Furthermore, there was a high percentage of missing data for calculating MEWS. We have excluded patients from analysis if two vital parameters other than temperature or mental status were missing. Also, we only included patients arriving between 10:00 hours and 18:00 hours potentially leading to selection bias. To improve the quality and clinical relevance of the data, future studies should also include cases where MEWS is found to be above the cut-off point, even if there are missing variables. Additionally, it is possible that the data were not missing at random. When a patient has normal vital signs during the first check, their vitals usually do not get monitored as frequently as when a patient initially has abnormal vital signs. Therefore, only including cases with known MEWS at all time points can cause a distorted view of the predictive performance of EWS in the ED, since there is a probability that patients with abnormal vital signs are disproportionately over-represented. It is important to record the full vital parameters set needed to calculate MEWS in clinical practice.

### Clinical implication

Implementation of a single standard time point for measurement of MEWS in the prehospital setting or ED is clinically not useful due to its moderate performance. However, patients with an increase of MEWS over time is highly correlated with the development of critical illness. Implementing standard repeated measurements in the acute care chain may result in better prediction of which patients are likely to become critically ill.

In conclusion, MEWS based on vital parameters measured at ED presentation has the highest accuracy in predicting the development of critical illness. However, performance is moderate and not significantly better compared with MEWS measured at other moments in the acute care chain. However, an increase in MEWS during the encounter is highly correlated with the development of critical illness. We, therefore, conclude that it would be valuable to assess MEWS over time, rather than only at a single moment.

## Data Availability

Data are available upon reasonable request.
